# Examining the ethical challenges in managing elder abuse: a systematic review

**Published:** 2019-06-15

**Authors:** Afsaneh Saghafi, Fatemeh Bahramnezhad, Afsaneh Poormollamirza, Ali Dadgari, Elham Navab

**Affiliations:** 1 *M.S, School of Nursing and Midwifery, Tehran University of Medical Sciences, Tehran, Iran. *; 2 *Assistant Professor, Department of Critical Care Nursing, School of Nursing and Midwifery, Tehran University of Medical Sciences, Tehran, Iran.*; 3 *M.S, School of Nursing and Midwifery, Tehran University of Medical Sciences, Tehran, Iran.*; 4 *Assistant Professor, Center for Health Related Social and Behavioral Sciences Research, Shahroud University of Medical Sciences, Shahroud, Iran.*; 5 *Associate Professor, School of Nursing and Midwifery, Tehran University of Medical Sciences, Tehran, Iran. *

**Keywords:** Elder abuse, Elder maltreatment, Ethics

## Abstract

Elder abuse is an increasingly intangible phenomenon that has created numerous ethical issues for care teams and caregivers. Although different studies have concentrated on various ethical issues regarding abuse, no study has arrived at a comprehensive conclusion. Therefore, the present study aimed to determine the existing ethical challenges in this context.

For this purpose, two researchers familiar with systematic search approach examined national and international journals on PubMed, Excerpta Medica Database (EMBASE), Scientific Information Database (SID) and similar databases between January and February 2017. They were able to find 116 articles that met the inclusion and exclusion criteria, and finally selected 15 articles based on the predesigned questions.

The findings were classified in five subtitles as follow: 1) the common definition of elder abuse, 2) a comprehensive legislation on elder abuse, 3) comprehensive ethical principles about elder abuse, 4) ethical considerations regarding patients without competency, and 5) reporting and sharing information about elder abuse. The study results revealed no common definition and no legislation about elder abuse, and also showed that health care providers’ observance of ethical principles depends on the ethical and legal conditions of the community.

Nowadays, elder abuse is a serious problem in many countries. Cultural and religious differences are the reasons for lack of a common definition and legislations, which comprises the biggest obstacle to protecting the rights of elderly people. It is clear that ethical principles should be respected as far as a person has competency. Furthermore, localization of clinical guidelines related to this issue leads to proper functioning of health care providers, especially nurses as the first line of treatment.

## Introduction

Abuse is among the most common challenging issues in both developed and developing countries around the world (1, 2). Presently, elder abuse is the most covert form of mistreatment that involves issues such as health, justice, ethics, and human rights (3). This phenomenon has been taken into consideration by World Health Organization (WHO) since 2002 (4). 

Different definitions have been provided for abuse over time (5). Elder abuse may refer to an act or absence of a proper act that will cause harm or suffering to an older person, and it happens in a relationship that normally requires trust, and may be performed only once or several times (1,2,6). 

While little information is available about elder abuse especially in developing nations, it is predicted to be on the rise in countries that experience the phenomenon of population aging. According to WHO estimations, one out of every six elderly people experiences abuse, and only 1 out of 24 abuse cases is reported (1). Since awareness of abuse is influenced by knowledge, expertise and preparedness of caregivers (3, 7), the care team and nurses as the first line of treatment are responsible to identify and report mistreatments and support vulnerable populations such as the elderly (8, 9). Elder abuse is an example of human rights and freedom violation (5) that leads to a serious loss of human dignity, independence and respect (6), and influences ethical principles such as autonomy, competency, beneficence, and non-maleficence  (10). 

Intervention in case of abuse is accompanied by ambiguity and ethical challenges, because lack of professional principles leads to personal, legal and ethical concerns (11). However, it is difficult for nurses and other members of the care team to perform a successful intervention for an elderly who is willing to stay in the abusive situation (12). Also, this phenomenon causes challenges for nurses and other care team members when legal commitments are not consistent with ethical principles (11). Questions and challenges in this context include: Are there any comprehensive ethical principles and regulations? Is it illegal to share the information and secrets of patients with the care team in order to reach a diagnosis or choose the appropriate intervention? Which ethical principle is violated in elder abuse? When is respect for autonomy not consistent with ethical principles? In what cases are beneficence and non-maleficence in conflict with the other ethical principles? In cases where the patient suffers from a cognitive disorder, what are the ethical considerations that need to be taken into consideration by the nurse? Should reporting elder abuse be a legal requirement? What ethical challenges will nurses face when the elderly are not willing to share information with the authorities? 

Abuse by a family member or intimate partner is complex, because the elderly may be struggling against social, cultural and religious aspects of life to live with abusive people (12). According to College of Nurses of Ontario (CNO), ethical conflicts and challenges emerge when two or several ethical values relevant to a particular situation necessitate conflicting measures (13). Elder abuse causes physical harm, depression, increased referral to hospitals, frequent hospitalizations, and increased mortality (1,2,14,15). It also creates problems such as job burnout and ethical distress for nurses and care team members (16), who should have a thorough and accurate understanding of the ethical concepts and challenges involved in elder abuse and decide on the best intervention (17). In view of the importance of this issue, this study aimed to examine the ethical challenges pertaining to elder abuse according to evidence-based ethical principles.

## Method

The present study was a systematic review to determine the ethical challenges involved in elder abuse and was conducted in 2017 by collecting related documents, articles, and sources. 

***Data sources and search method***

To find articles, national and international journals from databases such as PubMed, EMBASE, ProQuest Central, Web of Science, SID, Magazine Iranian (Magiran) and Psychology Information (PsycINFO) were examined. For this purpose, journals were searched using Persian and English keywords according to MeSH. Important keywords included “elderly”, “aged”, “abuse”, “neglect”, “mistreatment”, “ethics”, “ethos”, “moral”, and “autonomy”. In order to increase sensitivity, general Persian and English keywords such as “violence” and “senior” were also used in the search process. 

***Article selection criteria***

According to the inclusion and exclusion criteria, different articles available in the context of descriptive studies were examined. Since the nature of this issue is descriptive, articles with more evidence were selected. 

**Figure 1:**
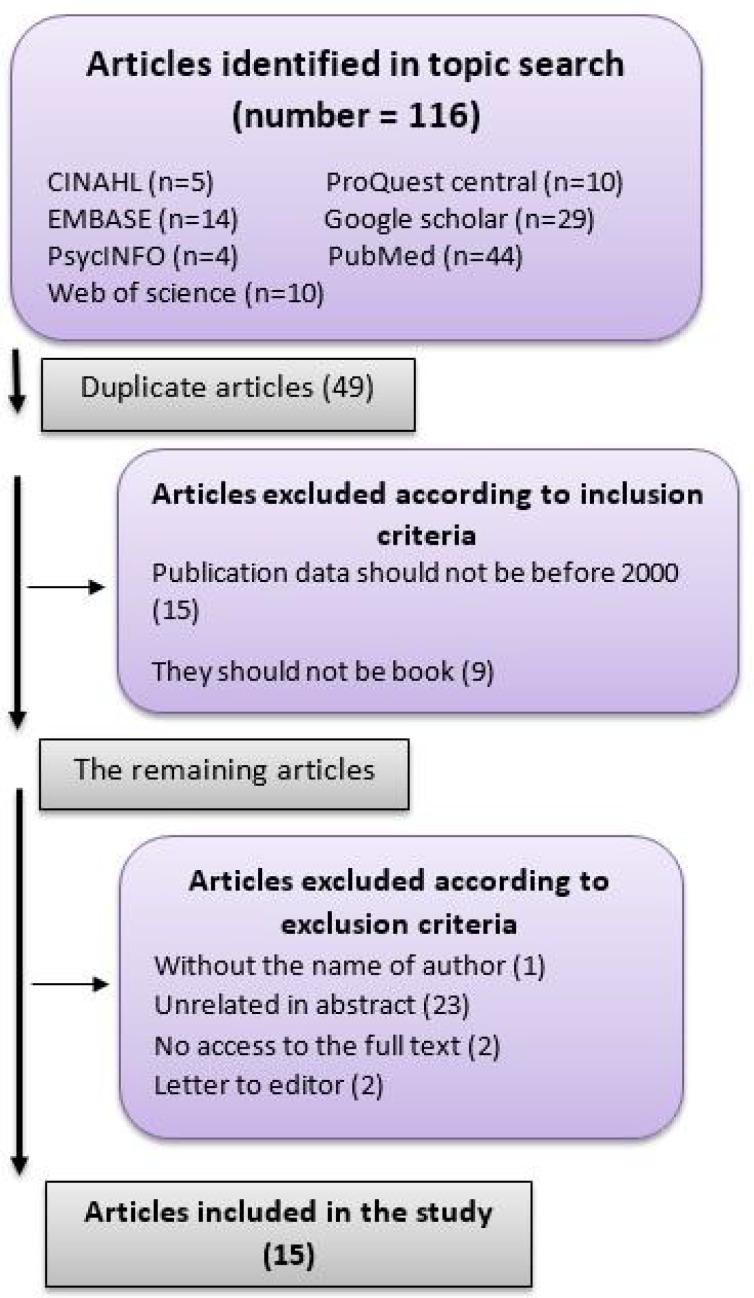
Article examination flowchart

The search was performed by two researchers familiar with systematic search approach between January and February 2017 according to the keywords and databases, and the details were documented. In this regard, 116 articles were found as seen in Figure 1.

Forty-nine articles were identified by EndNote to be duplicates and were therefore excluded from the study. According to the inclusion criteria, articles published after 2000 and those cases that had not been published were included in the initial examination. The abstracts were then studied by the two researchers in order to exclude unrelated articles, obtain the full texts for the ones that were related, and extract data for two of the articles whose full texts were not available. Finally, 15 articles were entered in the study to answer the following questions: Has a common definition been offered for elder abuse so far? Is there a comprehensive ethical set of regulations about elder abuse? What ethical principle is violated in elder abuse? When is the autonomy of elder people in conflict with ethical principles?

In order to minimize error, the required information was extracted according to the checklist (Table 1) by the two researchers and the results were matched. Checklist variables included title, author, year, journal, place and objective, definition of elder abuse, method, concepts, ethical issues, and clinical points. 

After collecting the data based on the checklist, data quality was assessed by two experts. In order to prevent bogus, names of journals and authors were eliminated and the data were given to two experts. The publication years of 15 articles were between 2000 and 2012, 9 articles had been authored in the United States, 1 in Canada, 3 in the UK, 1 in France, and 1 was a Korean-American article. Articles were descriptive, cross-sectional, and case study. No Persian article was found by searching these keywords.

## Results

Along with the proposed questions, the findings of the present study were classified in five subtitles as follow:

***1. The Common definition of elder abuse***

**Question:** Has a common definition been offered for elder abuse so far?

The researchers did not find any specific definition for the term. A study conducted in 2012 stated that concepts related to elder abuse are abstract and there is no widely accepted definition for it. This lack of definition poses challenging questions. US laws recognize cases such as physical abuse, neglect and financial abuse, but the definitions are not the same in various states and to all researchers and doctors (5). Another American study declares that all 50 states in the Unites States have laws concerning abuse, but the definitions for elder abuse and those who are covered by these laws are different (18).

In this regard, another study stated that in most US laws, physical, sexual, financial, and psychological/emotional abuse pertain to elder abuse, as does self-neglect, which is in concordance with the Older Adult Welfare Law (OAWL) definition of abuse (19). 

Adult Protective Services (APS) has identified three misconducts in connection with vulnerable elderly people: abuse, financial exploitation, and neglect. Abuse is defined as intentional harm to people. Financial exploitation is an illegal process of using vulnerable elderly people to obtain assets without their conscious consent. Neglect is the inability of an elderly person for self-care (self-neglect) or failure of a caregiver to provide appropriate care (20). Other studies have pointed to the definition provided by WHO that states “elder abuse consists of committing an inappropriate action in a trustful relationship that causes harm to an elderly person”, and four studies did not address the definition of abuse (21, 22, 23, 24). 

***2. A comprehensive legislation on elder abuse***

**Question**: Is there a comprehensive legislation about elder abuse?

Most US states combine elderly support laws and disabled adult laws. The laws are different in terms of classifications of abuse, but in general, elder abuse can be classified in five categories: physical abuse, psychological/emotional abuse, financial exploitation, neglect/self-neglect, and sexual abuse. Across the US, Elder Abuse Protection (EAP) laws share three common functions: 1) collecting the suspected abuse reports, statistical data management, and patients’ needs evaluation; 2) efforts to decrease or eliminate abuse risk for elderly people by providing instructions and interventions through referral to social services; 3) connection with courts and organizations that are responsible to execute the law in special cases (23, 25, 26). 

The results of Bergeron and Gray’s study in 2003 revealed three major differences between the laws in different states of the US. First, in some states, it is mandatory to report suspected elder abuse. The second difference is the definition of “elderly” provided by law. In order to limit interventions in the private lives of citizens, some states have restricted their laws to vulnerable elderly people. The third difference is related to the authorization given to EAP staff in some states to conduct studies on abuse and make interventions in cases where abuse has been proved. For example, some states ask the EAP staff to receive permission from the victim in all research steps unless the victim lacks competency, while other states do not (23). 

The National Association of Social Workers (NASW) and the Canadian Association of Social Workers (CASW) have specified values and principles as guidelines for the professional behavior of social services experts. These principles emphasize prioritization of the best interests of patients and protection of those who lack competency for decision-making. They also require that confidentiality of information be taken into consideration, which may be in conflict with abuse reporting responsibilities (11). Also in January 2004, OAWL established stable laws to offer protection services for elderly people such as 24-hour emergency phone lines to report abuse, standards to issue certificates for protective facilities, and professional standards to care for elderly people (19).

***3. Comprehensive ethical principles about elder abuse***

**Question:** Are there any comprehensive ethical principles about elder abuse?

A study conducted in the Unites States pointed out that when disclosing information, psychologists should use American Psychological Association (APA) codes (5, 27). In this regard, another study proposed that psychologists consider ethical commitments in addition to the legal aspects of abuse reporting. 

Reporting suspected cases according to APA ethical codes should be in line with beneficence, non-maleficence, and respect for human rights and dignity. Code 4/02 is related to privacy limitation and code 4/05 pertains to disclosure of information regarding elder abuse. If the elderly suffers from cognitive disorders, psychologists should try to maintain the confidentiality of their information and contact legal authorities. According to code 4/02, they are allowed to disclose information in suspected abuse cases even without the patient’s consent. According to code 4/05, psychologists are allowed to share the private information of patients with the latter’s families or with other experts to protect patients against harm (18). 

In a 2009 study by Doe et al., similarities and differences between the protection systems of Korea and the United States were investigated. The two countries are similar in that they both have national laws to prohibit elder abuse, and legal definitions include abuse and neglect. In addition, laws necessitate mandatory reporting by experts. As for the differences, in the United States, federal laws are executed by different federal systems that implement both the methods of reporting abuse and referral. Emergency cases enter triage from the very beginning and a protection plan is designed for abuse cases and other forms of mistreatment. Laws prosecute the guilty person and punishments are specified for failure to report. However, in Korea, the law is enforced by a centralized system that only implements the reporting of abuse. Emergency cases are triaged within 12 hours and care plan only exists for abuse cases. These laws allow the patient to file a civil complaint about abuse, but they do not specify any punishment for failure to report (19).

**Question: **What ethical principle is violated in elder abuse?

Findings showed that the principle of respect (dignity and autonomy) is almost violated in psychological abuse, and the principle of non-maleficence in cases of neglect and physical or financial abuse (28). In most cases, autonomy may be violated because self-neglect, beneficence and non-maleficence may come before it, and as for self-neglect, caregivers can delay the required interventions as far as possible  (10).

*Autonomy*

Autonomy includes independent decision-making without any limitation, and respect for independence is a professional commitment (29). According to the American Nurses Association (ANA) autonomy not only means respect for patients’ decision-making, but also for the decision-making method (30), and patients have the right to participate in making decisions related to themselves (31). Another study stated that social workers are responsible for creating a balance between patients’ rights and the principle of autonomy, which aims to protect vulnerable populations. Nevertheless, in NASW, it is pointed out that social workers should have authority over patients’ right to autonomy when potential and actual actions of patients cause serious, predictable and unpredictable risks for themselves and others (11, 32). Also, another was consistent with the above-mentioned points and stated that autonomy to maintain independence is acceptable only as far as it is reasonably and ethically possible  (10).

Negative autonomy emerges when the elderly prevents services and the caregivers accept this behavior, which is indeed a kind of neglect. Prevention is acceptable if the person has the capability for decision-making and his or her mental capacity is approved (33). 

*Beneficence and non-maleficence*

Beneficence and non-maleficence are both based on ethical commitment toward others, and while the former focuses on the well-being of others, the function of the latter is to avoid harming them (29). Together, the two aspects provide more comprehensive principles to devise measures against elder abuse. For example, if a nurse considers a threat or damage serious and is convinced that measures are necessary to prevent harm, he or she will report the abuse to APS. Therefore, the nurse may use the principle of beneficence to promote health and ensure the best interests of the patient  (10). 

*Justice*

In the 15 papers that were examined in this study, the principle of justice had not been mentioned. 

***4. Ethical considerations regarding patients without competency***

**Question: **In cases where the patient lacks competency, what ethical considerations should be taken into account by the nurse or the care team?

A study entitled “Capacity for decision-making in Alzheimer's disease: selfhood, positioning and semiotic people” showed that capacity and competency are often used interchangeably. Capacity has dimensions such as decision-making, self-care, self-protection, and execution. According to experts, competency can fluctuate. In dementia, there is memory impairment, but personality, values and long-term memory stay intact. Dementia patients can be extremely vulnerable under undesirable conditions and their right of decision-making for different aspects of their life is unfairly influenced (34). 

Dementia diagnosis is not the only criterion for lack of competency (35, 36). For competent elderly people who expose themselves or others to harm, the caregiver may decide to work in support of beneficence, trying to achieve the best long-term results for the patient. It is noteworthy that one should consider interdisciplinary interventions, ethical principles, and cultural and gender differences when trying to determine decision-making capacity by means of valid and reliable measurements.

Dick suggests that nurses should note the cultural beliefs and patterns of adaptation of family members who neglect an elderly person’s personal and environmental health requirements rather than consider it a pathological finding (37). Nevertheless, according to most studies, when the elderly is incapable of decision-making due to cognitive impairment, the task can be left to another person. This person should support the patient and be aware of his/her needs. Most regional councils for elderly issues provide care services in which a group of competent people function as decision-makers for elderly people (5). In cases where decisions are related to the patient’s capacity, consultation with a group of interdisciplinary experts is necessary and the decisions should eventually be in favor of the elderly people  (10).

***5. Reporting and sharing information about elder abuse***

**Question: **Is it illegal to report abuse and share the information of elderly people? 

Respect for confidentiality and trust is one of the most important ethical principles that has to be taken into consideration by caregivers. However, the results of one study indicate that in cases where a serious harm is caused, the care team can disclose information without obtaining consent (11). These findings were confirmed by another study, which presented statements with similar wording. For example, in the United States, if a therapist is suspicious about abuse, he/she should report it to authorities such as APS despite his/her concerns over the patient’s privacy (26). Also, according to code 4/05, psychologists are allowed to disclose patients’ private information to protect them against harm (17). However, they should try to engage the patient in the reporting process and only report relevant data to observe the privacy of the patient as far as possible (26).

**Table 1:** Characteristics of the reviewed articles on the ethical challenges in caring for elder abuse

Objective	Type of Study	Country	Year	Resource
**Updating moral considerations ** **regarding elder abuse in psychological ** **domain Providing recommendations to ** **improve problem solving related to ** **elder abuse**	descriptive	The United States	2012	Scheiderer EM(5)
**Forms of elder abuse in nursing homes ** **Reasons for elder abuse in nursing ** **homes Which ethical principles are ** **violated in nursing homes?**	descriptive	The United States	2011	Bužgová R (28)
**Discussion about important ethical ** **perspectives on self-neglect among ** **elderly people**	descriptive	The United States	2011	Mauk KL (10)
**Examining abused people according to ** **ethical codes and guidelines to enable ** **psychologists to solve the problems of ** **these people** **Helping to resolve challenges to ** **reporting these cases and specifying ** **certain standards for reporting.**	descriptive	The United States	2011	Zeranski (18)
**Solving the complications arising from ** **elder abuse ** **Concentrating on informing social ** **services experts about challenges ** **resulting from ambiguity in laws and ** **guidelines**	descriptive	Canada	2010	Donovanet (11)
**Examining the gap between the ** **existing laws about elder abuse, and ** **implementing them** **Examining protection of elderly people ** **against elder abuse**	descriptive	England	2010	Ash(21)
**Examining the existing texts about ** **elder abuse ** **Suggesting preventive measurement to ** **decrease the incidence of abuse**	descriptive	England	2010	Formanet (26)
**Examining the relationship between ** **cultural values such as individualism ** **and identification of the ethical issues ** **and consequences of policymaking; ** **Discussing about similarities and ** **differences in the reporting system**	descriptive	Korean- American	2009	Doe (19)
**Examining women’s understanding of ** **different economic and social ** **conditions regarding elder abuse and ** **the ethical problems in support ** **systems**	descriptive	The United States	2009	Dakin (38)
**Determining who the people ** **experiencing abuse are, how they are ** **identified, and which professionals are ** **trying to resolve these conditions?**	descriptive- cross- sectional	France	2006	François (39)
**Describing an ethical decision-making ** **model according to an empowerment ** **framework for care service providers**	descriptive	The United States	2006	Koenig (22)
**Proposing the perspective of ** **psychologists on elder abuse, and the ** **psychological and ethical issues ** **regarding this subject**	descriptive	The United States	2006	Beaulieu (33)
**Stating the reasons for abuse according ** **to the statements of social workers in ** **England**	descriptive	England	2004	Wilson (24)
**Discussing ethical concerns about ** **reporting or failure to report abuse ** **Providing recommendations to help ** **service providers in the decision-** **making process**	descriptive- case study	The United States	2003	Bergeron (23)
**Establishment of a standard ethical ** **model to deal with elder abuse**	descriptive	The United States	2000	Johnson (40)

## Discussion

Concepts related to elder abuse are complex and abstract, and therefore no common definition has been offered for this phenomenon (5). In most cases, the word abuse is replaced by maltreatment and mistreatment (41). One reason for lack of a common definition is related to cultural and religious differences among societies. Accordingly, people from different races have their own definition of abuse and its types based on their regional priorities (41, 42). 

Care team members need a clear definition to identify and prevent elder abuse (43). When a suitable definition does not exist for the phenomenon, accurate statistics cannot be obtained, which makes it impossible to identify and report abuse; consequently, the prevalence of this phenomenon will remain obscure. Therefore, measures related to elder abuse should be specific to each region where authorities function as the main foundations. 

Another duty of governments includes establishment of regulations and ethical principles related to elder abuse. Since comprehensive ethical and legal regulations have not been developed and there are many ambiguities and conflicts, abuse cases are not reported due to the inability of the care team to interpret the ethical and legal codes (5). 

Based on the existing evidence, elder abuse is a general issue in Italy where different policies and laws exist for the phenomenon and lack of a national and comprehensive strategy is tangible. While certain laws are in place for child protection, there are no specific regulations for elderly people. 

Lack of legislation is the biggest obstacle to protecting the rights of elderly people in many countries. Constitutions normally emphasize rights, freedom, dignity and equality for all people, with the main emphasis on the latter. Elderly people may not be able to defend their rights. Therefore when they become dependent, they may be vulnerable to abuse and misconduct (44).

The first federal law on elder abuse was codified as “The Elder Justice Act” in March 2010 including the following main articles: (a) formation of the elder justice council to suggest recommendations related to federal, regional and private agencies involved in elder abuse; (b) formation of a council to plan strategic programs on elder justice; (c) budget provision; (d) founding and supporting legal centers; (e) provision of budget for long-term treatment programs; (f) financial assistance to improve long-term treatment programs for staff; and (g) budget provision for national institutes (45, 46).

Moreover, laws related to APS aim to control elder abuse in home environments or institutes. Depending on the type of law, in each country there is a reporting system for elder abuse and social services provisions to support victims and change the conditions. Nevertheless, the aforementioned laws are mostly related to abuse of adults with disabilities and vulnerabilities rather than elder abuse (47).

Therefore, the availability of ethical principles and laws helps the care team deal with abuse. Ethical principles are laid down by experts to protect the profession, and social workers will be directed to implement the pertinent activities by standard establishment. This helps professionals defend themselves against Ethics Supervisory Boards as well (47). 

Sometimes professionals deal with elderly people who experience conditions not directly specified by ethical standards, and this creates challenges (48). In such cases, members of the care team face controversial ethical and legal conditions in action. Ethical principles concentrate on rights and commitments against ethical challenges and specify what care team members should do in order to observe these principles. However, they may refrain from performing an ethical task for fear of the consequences, and may not implement any type of intervention in cases of abuse because they are concerned about violation of the patients’ rights (11). 

Ethical principles are taught theoretically and when complications arise, care team members have a tendency to neglect ethical teachings, believing that theoretical teaching is one of the factors that complicate elder abuse (24). It is necessary for those who deal with elderly people to be aware of the professional, legal and ethical issues of elder abuse. In order to stay committed to caregiving values, the care team should have an effective performance and provide safety for elderly people and at the same time respect their dignity. 

Important ethical principles that are violated in abuse are autonomy, beneficence, non-maleficence, and justice. This phenomenon not only involves elderly people, but also other vulnerable classes of the society such as women, children, people with disabilities, and people who suffer from mental disorders. Abuse creates challenges for the care team and puts them in dilemma. The question that now arises is: should the rights and freedom of an elderly person who has been abused be violated out of concern for his or her safety? In truth, the dual commitment of staff and other caregivers may violate the patient’s right to autonomy. 

One study showed that professionals who are responsible for protecting patients should keep in mind that when an elderly person decides to reject services (autonomy) and continue to live in threatening conditions, logical thinking under critical circumstances will be a necessity (11). In a 2003 study by Healy the responses to a self-assessment by social services staff to ethical dilemmas concerning the decision-making capacity of elderly patients suffering from cognitive impairment are reported. In the section pertaining to house safety conditions, participants stated that they were faced with conflict when they had to force the person to go somewhere safe or respect his/her decision to continue in the existing conditions. In such cases, social workers face a decision-making challenge: to refrain from intervention and respect the person’s autonomy, or try to prevent harm to the person and introduce the culprit to the judiciary system (49). 

However, discussion about ethical principles is complex, because they are based on the culture in each society. Autonomy should be respected as far as the person has competency and poses no risk for people and the community. The principles of beneficence and non-maleficence are no exception. When a patient’s decision jeopardizes individual and social interests, the two principles of beneficence and autonomy are in conflict, because respect for one principle violates the other. In these cases, the care team should select the best functional option according to regional interests, consultations and guidelines. 

Another controversial issue relating to abuse is the principle of confidentiality. Confidentiality is one of the important principles ruling the relationship between patients and the care team, and one justification for it is respect for patient’s autonomy. The principle of autonomy emphasizes the patient’s right over all stages of life. Therefore, one’s personal information belongs to oneself and no one should be aware of it without permission. If confidentiality is violated, the patient’s autonomy will be violated too. 

However, reporting abuse is another challenging issue. Discussion about elder abuse reporting is not limited to the social context, but it necessitates awareness about medication, law execution, and social services. All professionals who have relationships with elderly patients regarding treatment and law execution should assume the ethical responsibility to protect them against harm. 

Rodriguez et al. conducted interviews in 2006 with primary health-care providers. They concluded that in Los Angles, physicians experience a conflicting relationship between life quality and obligatory reporting laws. Most respondents believed that the effects of abuse result in both improvement and harm for the patient, and experts’ tendency to promote patients’ life quality may decrease due to frequent reports (50). 

The ethical principles of the American Counseling Association (ACA) emphasize the mandatory nature of consultation with other experts when counselors are not sure whether the case meets the reporting criteria or not. It should be mentioned that professionals should not only report suspected abuse, but they should be familiar with laws and regulations related to reporting, so that if a problem occurs, they can report it effectively (23). 

Opponents of mandatory reporting argue that this type of reporting violates autonomy where the privacy of people is compromised (38). Evidence shows that social workers in Korea choose not to execute mandatory reporting because they feel that by modifying the conditions that cause abuse, family members can participate in providing care for the elderly at home. Social workers believe that providing care at home, improving the relationship between the elderly and their families and intermediation role provide a better cultural option for the elderly (19). There are concerns about receiving services from all ethnic groups. Studies on services showed that elderly people from various racial groups prefer to receive assistance from family members and friends to solve all types of problems except health and financial problems (23, 51). However, the mandatory reporting law should observe the dignity and privacy of people.

Now, the question is, what is the objective of the care team in reporting suspected abuse cases? The objective should not be to obtain or complete the statistics of abuse cases, but the principles of beneficence and non-maleficence should be prioritized. The reporting system is unique in every country and follows the ethical principles and laws of that country. Mandatory reporting is valued when there are protective systems and laws to help the elderly and rescue them from trouble. In countries where these laws do not exist, however, mandatory reporting does not promote a person’s quality of life, and even increases the gap between that person and his/her family members who have committed abuse. Since a support system is non-existent in these countries, the elderly has to continue to live with the abusive family member(s), who may do more harm to the elderly, and even cause their death, because no education, reward or punishment system is in place. 

As for the competency of the elderly, the obvious point is that despite the availability of instruments such as the Mini-Mental State Examination (MMSE) that examines cognitive capacity, researchers should fully examine the cultural concerns associated with environmental and lifestyle patterns    (52). 

Finally, for decision-making the care team can benefit from the communication ethics approach, which is a participatory method comprising several stages: (a) the defense stage to clarify conditions and issues; (b) consultation; and (c) negotiation that leads to agreement (40). 

***Iranian Ethical Challenges***

The definition of elder abuse varies in different societies. According to an Iranian study by Heravi et al. conducted in 2013, elder abuse may be defined as an act or absence of a proper action by family members or relatives, which may happen once or several times, and can cause harm or distress to an older person (53).

Comprehensive ethical and legal regulations have so far not been developed in Iran. According to investigations by a researcher in Iran, no specific law exists about reporting and handling elder abuse. The Secretariat National Council of the Elderly in Iran, established in 2004, is the only organization that is active in various fields related to the elderly, but it seems that no significant activity has been performed regarding elder abuse. Also, Social Emergency has been active in Iran since 2007 to prevent harms and also provide services in connection with elder abuse, but they have not mentioned any specific ethical principles for managing elder abuse, its identification and referral. It is clear that availability of ethical principles and laws can help the care team deal with abuse.

In chapter three of the Constitution of the Islamic Republic of Iran, the importance of autonomy and human dignity has been addressed. According to the constitution, respect for human dignity is a principle accepted by the Islamic Republic of Iran, obliging the government to provide care services to everyone (54). Accordingly, care services should be based on respect for rights and dignity of patients. Accordingly, the patient should choose and decide freely. Respect should be provided for the patient while receiving care services based on respect for patients’ privacy and confidentiality of information. In this regard, a study by Davis et al. showed that the possibility of decision-making is vital for elderly people, and lack of autonomy leads to depression (51).

Based on our Iranian-Islamic beliefs, confidentiality means trust, and it has to be protected. Sharing personal information with others indicates betrayal. Also, according to Article 648 of the Islamic Penal Code, disclosure of patients’ secrets is illegal except when personal or public interest is at risk (55). 

In Iran, there is no particular penal code for elderly people, but according to Article 596 of the Islamic Penal Code on financial abuse, the abuser will be sentenced to 16 months to 2 years of imprisonment and payment of a fine. If the abuser is the guardian of the victim, in addition to the fine, the abuser will be sentenced to 3 to 7 years of imprisonment (56). 

Thus, according to the above-mentioned points, elder abuse is a phenomenon that needs more attention from the government.

## Conclusion

Abuse is a serious problem among elderly people. Although the care team is responsible for the support and promotion of the independence of elderly people, ethical challenges are the result of unsatisfactory performance of the care team and people who endanger themselves or the others. 

In decision-making about measures related to elder abuse, ethical principles such as autonomy, beneficence, non-maleficence and competency should be taken into consideration. The care team should protect the autonomy of elderly people and consider their health and welfare. However, they should not impose their beliefs regarding living environments or social decision-making on the elderly. 

As far as possible, the relationship between elderly people and family caregivers should be promoted. At the same time, protecting elderly people will not be possible unless the society can help them maintain their independence outside of the family system. This will allow the elderly to enjoy a positive relationship with members of their family who are also their caregivers. The achievement of these goals necessitates long-term coordination between services and institutes. 

Some measures that can be effective in this context include: devising appropriate instructions for the care team, particularly nurses who deal with abuse issues; communication and consultation with other service providers while observing privacy and autonomy; commitment to follow instructions; observing ethical considerations regarding abuse; and conducting empirical studies. 

Nurses as the first line of treatment and other care team members have an important role in this respect. Compilation or localization of clinical guidelines for the care team not only influences their perspectives into ethical issues, but also helps them perform properly and select the best functional option. Finally, clinical guidelines support the behavior and performance of the care team and serve as a criterion to assess the caregiving quality.

## Notes:

**Citation to this article:**

Saghafi A, Bahramnezhad F, Poormollamirza A, Dadgari A, Navab E. Examining the ethical challenges in managing elder abuse: a systematic review J Med Ethics Hist. 2019; 12: 7.
